# Risk factors for clinical progression in patients with pulmonary *Mycobacterium avium* complex disease without culture-positive sputum: a single-center, retrospective study

**DOI:** 10.1186/s40001-023-01152-0

**Published:** 2023-06-08

**Authors:** Mizu Nonaka, Masashi Matsuyama, Chio Sakai, Sosuke Matsumura, Naoki Arai, Masayuki Nakajima, Takefumi Saito, Nobuyuki Hizawa

**Affiliations:** 1 Department of Respiratory Medicine, National Hospital Organization Ibarakihigashi National Hospital, Ibaraki, Japan; 2 Department of Respiratory Medicine, Faculty of Medicine, University of Tsukuba, Ibaraki, Japan; 3 Department of Pulmonary Medicine, Faculty of Medicine, University of Tsukuba, 1-1-1 Tennoudai, Ibaraki, Tsukuba 305-8575 Japan

**Keywords:** Nontuberculous mycobacteria, *Mycobacterium avium* complex

## Abstract

**Objectives:**

Limited data are available on the progression of pulmonary *Mycobacterium avium* complex (MAC) disease without culture-positive sputum. The aim of this study was to identify the risk factors associated with clinical progression of pulmonary MAC disease diagnosed by bronchoscopy.

**Methods:**

A single-center, retrospective, observational study was conducted. Pulmonary MAC patients diagnosed by bronchoscopy without culture-positive sputum from January 1, 2013, to December 31, 2017 were analyzed. Clinical progression after diagnosis was defined as having culture-positive sputum at least once or initiation of guideline-based therapy. Then, clinical characteristics were compared between clinically progressed patients and stable patients.

**Results:**

Ninety-three pulmonary MAC patients diagnosed by bronchoscopy were included in the analysis. During the 4-year period after diagnosis, 38 patients (40.9%) started treatment, and 35 patients (37.6%) had new culture-positive sputum. Consequently, 52 patients (55.9%) were classified into the progressed group, and 41 patients (44.1%) were classified into the stable group. There were no significant differences between the progressed and the stable groups in age, body mass index, smoking status, comorbidities, symptoms, or species isolated from bronchoscopy. On multivariate analysis, male sex, monocyte to lymphocyte ratio (MLR) ≥ 0.17, and the presence of combined lesions in the middle (lingula) and lower lobes were risk factors for clinical progression.

**Conclusions:**

Some patients with pulmonary MAC disease without culture-positive sputum progress within 4 years. Therefore, pulmonary MAC patients, especially male patients, having higher MLR or lesions in the middle (lingula) and lower lobes might need careful follow-up for a longer time.

## Introduction

The incidence and prevalence of pulmonary nontuberculous mycobacterial (NTM) disease are increasing worldwide [1–3]. The reason for this is not clear, but one possible factor is the increased attention paid to this disease since the introduction of the guidelines in 2007 [1, 4]. In addition, it is expected that many cases with early pulmonary NTM will be diagnosed, especially in areas where health checkup systems are widely available [5]. The current ATS/ERS/ESCMID/IDSA guideline for pulmonary NTM disease established clinical, radiological, and microbiological criteria for diagnosis [6]. The microbiological criteria include the isolation of NTM in at least two sputum cultures or at least one specimen culture from bronchoscopy. Therefore, more patients will be diagnosed by bronchoscopy with early disease without culture-positive sputum.

Despite the high mortality rate of patients with NTM, there is little evidence of the importance of early diagnosis by bronchoscopy [7, 8]. This is because we have insufficient knowledge of the prognosis of early disease and the risk factors for progression. In particular, only a few retrospective studies have shown the progression of pulmonary NTM disease diagnosed by bronchoscopy. Research in Taiwan has shown that the risk factors for radiological progression in pulmonary NTM patients without positive acid-fast bacilli (AFB) cultures of sputum include male sex, low body mass index (BMI), inhaled corticosteroid (ICS) use, and positive AFB smear (2+ or more) by bronchoscopy [9]. A Korean study showed no clear difference in disease progression between patients diagnosed by bronchoscopy and those diagnosed by sputum [10]. However, these studies included other species of bacteria besides *Mycobacterium avium* complex (MAC), such as *Mycobacterium abscessus* and *Mycobacterium kansasii*. Because the species distribution of NTM isolates varies widely by region, and MAC is the most common species in many locations, with particularly high detection rates reported in Australia and Japan [11], the present study focused specifically on pulmonary MAC disease.

Several studies have shown that the complete blood count (CBC) and peripheral leukocyte fraction, including the monocyte to lymphocyte ratio (MLR), might be potential biomarkers for mycobacterial infection [12–15]. However, these CBC tests lack sufficient data to be useful for identifying pulmonary MAC disease or for predicting progression. Serum antibody against glycopeptidolipid (GPL)-core IgA antigen is one of the serological biomarkers used for both diagnosis and assessment of disease activity in pulmonary MAC disease [16]. This GPL-core IgA antibody is widely used in Japan to test patients with suspected pulmonary MAC disease.

The present study aimed to investigate the clinical characteristics related to clinical progression, including CBC, MLR, and serum antibody against GPL-core IgA antigen, in patients with pulmonary MAC disease diagnosed by bronchoscopy.

## Materials and methods

### Study design

This retrospective study was conducted in National Hospital Organization Ibarakihigashi Hospital. This study was approved by the Institutional Review Board of Ibarakihigashi National Hospital (No. 2022-001). The requirement to obtain informed consent was waived, because this was a retrospective study. Patients meeting the following criteria were identified from the medical records: (1) newly diagnosed with pulmonary MAC disease by bronchoscopy from January 2013 to December 2017; and (2) met the ATS/ERS/ESCMID/IDSA criteria [6]. Of these patients, those with the following conditions were excluded: (1) duration of follow-up less than 4 years after bronchoscopy; and (2) sputum mycobacterial culture was positive at diagnosis. The primary outcome was clinical progression of pulmonary MAC disease within 4 years after bronchoscopic diagnosis. Clinical progression after diagnosis was defined as one or more culture-positive sputum samples or clinical and radiographic deterioration leading to initiation of guideline-based therapy (GBT) [6]. Patients who never had a positive sputum culture during the 4-year period and were not started on GBT were defined as the stable group. During the 4-year period, attending physicians determined the interval of sputum culture examinations and the time of treatment initiation, depending on the symptoms or presence of radiological deterioration. In some patients, both the development of culture-positive sputum and the initiation of GBT were observed, and whichever occurred earlier was taken to be the time of disease progression.

Medical records, radiological findings, and microbiological findings were retrospectively reviewed to assess risk factors for clinical progression. Since a previous report of the long-term natural history of stable pulmonary MAC disease indicated that approximately 60% of patients with pulmonary MAC disease begin treatment within 3 years, an observation period of 4 years was set in the present study [17].

### Clinical assessment

Baseline characteristics at the time of bronchoscopic diagnosis were obtained from patients’ medical records, including age, sex, BMI, smoking status, comorbidities, presence of respiratory symptoms, and laboratory examination results. Laboratory data were collected, including white blood cell counts, neutrophil counts, lymphocyte counts, monocyte counts, eosinophil counts, basophil counts, MLR, albumin, and GPL-core IgA antibody. MLR was defined as the monocyte count divided by the lymphocyte count. A GPL-core IgA antibody titer ≥ 0.7 U/mL was considered positive. All blood tests were performed immediately prior to bronchoscopy.

### Radiological assessment

Two pulmonologists reviewed the computed tomography (CT) images at the time of bronchoscopic diagnosis. Radiographic findings due to NTM lesions, including bronchiectasis, cavities, infiltrates, and nodules, were evaluated. Patients were categorized as having fibrocavitary (FC) type or nodular bronchiectatic (NB) type based on these findings. Furthermore, patients with type NB were classified into three types according to the predominant imaging pattern that accounted for at least 50% of the lesions: granular/nodular shadow-predominant, bronchiectasis-predominant, and consolidation-predominant. The locations of the lesions and the number of involved lobes were also analyzed. The locations of the lesions were evaluated in separate sections: upper, middle (lingula), and lower lobes, with the lingula considered a separate lobe.

### Microbiological assessment

Microbiological information was obtained by bronchial washing specimens for assessment at the time of diagnosis, and sputum specimens were obtained for assessment of progression. All bronchial washing specimens at diagnosis were inoculated into Mycobacteria Growth Indicator Tube (MGIT) liquid media, Ogawa solid media, blood agar media, chocolate agar media, and bromothymol blue (BTB) agar media. Sputum specimens were inoculated into MGIT liquid media and Ogawa solid media. The MGIT liquid media and Ogawa media were observed until 6 weeks and 8 weeks, respectively. The bacterial species were identified using COBAS^®^ TaqMan^®^ 48 testing (Roche Diagnostics, Basel, Switzerland), TRCReady^®^-80 (Tosoh Bioscience, Tokyo, Japan), and Vitek2^®^ (Sysmex bioMérieux, Tokyo, Japan). Species, AFB smear, and time to culture-positive at diagnosis were evaluated. The shorter incubation time of the two media was defined as the time to culture positivity.

### Statistical analysis

Categorical variables are presented as numbers (percentage) and were compared using the Chi-squared test. Continuous variables are presented as medians (interquartile ranges) or means (standard deviation) according to the distribution, and they were compared using the Mann–Whitney *U* test, Kruskal–Wallis test, and Student’s *t* test, as appropriate. Logistic regression analysis was performed to identify independent factors for clinical progression.

Because no reference value for MLR has been established in healthy subjects, a cutoff value of 0.17, the value of MLR at which Log_10_MLR was the mean value of the stable group, was used in this study (Log_10_ [0.17] = −0.77).

Variables with a *p* value < 0.1 on univariate analysis, as well as age and sex, were included in the multivariate analysis. Adjusted odds ratios with 95% confidence intervals and* p* values were calculated. To analyze the cumulative rate of clinical progression after diagnosis, the Kaplan–Meier method with the log-rank test was used. Receiver-operating characteristic (ROC) curve analysis was performed to assess the predictive value of MLR to identify disease progression. All tests of significance were two-sided, and *p* values < 0.05 were considered significant. Pairwise deletion was used to handle missing data. All statistical analyses were performed with IBM SPSS 28.0 (IBM Corp., Armonk, NY, USA).

## Results

### Patients’ characteristics

The clinical characteristics of the patients at diagnosis are shown in Table 1. Ninety-three pulmonary MAC patients diagnosed by bronchoscopy were included in this study. During 4-year follow-up after diagnosis, 38 (40.9%) patients started GBT, and 35 (37.6%) patients produced culture-positive sputum at least once. Of the 93 patients, 52 (55.9%) were classified into the progressed group, and 41 (44.1%) were classified into the stable group. The median age of the patients was 70 years (64–75 years), and 21 (22.6%) of them were male. The major comorbidities were hypertension (24.7%), history of pulmonary tuberculosis (7.5%), uterine myoma (6.5%), and asthma (6.5%). Table 2 shows bacterial profiles from bronchoscopic specimens. MAC species isolated at diagnosis included *Mycobacterium avium* (67.7%) and *Mycobacterium intracellulare* (31.2%). Isolated bacterial species other than MAC from the specimens were also evaluated as co-infections. *Veillonella species* (57.0%) were the most common co-infecting species.

**Table 1:** Patients’ baseline characteristics

Variables	Overall (*N* = 93)	Stable group (*N* = 41)	Progressive group (*N* = 52)	*P* value
Age (year)	70 (64–75)	70 (64–75)	70 (64–76)	0.650
Male sex	21 (22.6)	5 (12.2)	16 (30.8)	**0.033**
Body mass index (kg/m^2^)	20.8 ± 2.4	21.2 ± 2.7	20.5 ± 2.1	0.172
ICS user	6 (6.5)	2 (4.9)	4 (7.7)	0.583
Smoking status				0.711
Never smoker	72 (77.4)	31 (75.6)	41 (78.8)	
Ex-smoker	21 (22.6)	10 (24.4)	11 (21.2)	
Current smoker	0 (0)	0 (0)	0 (0)	
Comorbidities				
Hypertension	23 (24.7)	13 (31.7)	10 (19.2)	0.166
Asthma	6 (6.5)	2 (4.9)	4 (7.7)	0.583
History of pulmonary tuberculosis	7 (7.5)	2 (4.9)	5 (9.6)	0.390
Diabetes mellitus	5 (5.4)	4 (9.8)	1 (1.9)	0.096
Autoimmune disease	2 (2.2)	2 (4.9)	0 (0)	0.107
Sinusitis	5 (5.4)	2 (4.9)	3 (5.8)	0.850
Uterine myoma	6 (6.5)	2 (4.9)	4 (7.7)	0.583
GERD	2 (2.2)	1 (2.4)	1 (1.9)	0.865
Symptoms				
Cough	21 (22.6)	9 (22.0)	12 (23.1)	0.897
Sputum	15 (16.1)	4 (9.8)	11 (21.2)	0.138
Hemoptysis	2 (2.2)	0 (0)	2 (3.8)	0.204
Weight loss	3 (3.2)	1 (2.4)	3 (3.2)	0.703
Fever	1 (1.1)	0 (0)	1 (1.9)	0.372
Laboratory exams				
Positive for anti-GPL–core IgA antibody *	49 (53.8)	21 (52.5)	28 (54.9)	0.820
WBC (/μL)	5400 (4600–6500)	5200 (4500–6400)	5450 (4700–6500)	0.642
Segment (/μL)	3393 (2861–4164)	3202 (2840–4132)	3470 (2946–4184)	0.262
Lymphocyte (/μL)	1421 (1108–1818)	1552 (1235–1836)	1322 (1099–1673)	0.093
Monocyte (/μL)	270 (231–342)	256 (202–301)	285 (239–350)	**0.018**
Eosinophil (/μL)	81 (50–138)	81 (50–138)	81 (50–139)	0.997
Basophil (/μL)	21 (13–38)	22 (20–38)	20 (12–33)	0.265
Alb (g/dL) ^†^	4.3 (4.1–4.6)	4.4 (4.2–4.5)	4.3 (4.0–4.6)	0.218
MLR	0.18 (0.14–0.25)	0.15 (0.13–0.20)	0.20 (0.17–0.28)	**<0.001**
Log_10_MLR	−0.73 ± 0.17	−0.77 ± 0.18	−0.67 ± 0.15	**0.002**
MLR ≥ 0.17	57 (61.3)	17 (41.5)	40 (76.9)	**<0.001**
Reason for clinical progression				
Treatment initiation	38 (40.9)	0 (0)	38 (73.1)	**<0.001**
Positive sputum culture within 4 years after diagnosis	35 (37.6)	0 (0)	35 (67.3)	**<0.001**

ICS, Inhaled corticosteroid; GERD, Gastroesophageal reflex disease; GPL, Glycopeptide lipid; MLR, Monocyte to lymphocyte ratio. Categorical variables are represented as absolute numbers (%). Continuous variables with normal distributions are shown as means ± standard deviation. Continuous variables with non-normal distributions are shown as medians and interquartile range. Categorical variables were analyzed using the Chi-squared test. Continuous variables were analyzed by the Mann–Whitney *U* test or Student’s *t* test as appropriate. *P* values are for comparisons between stable group and progressive group. Bold indicates significance (*P* < 0.05)

*Positivity of anti-GPL–core IgA antibody are shown. *N* = 91, 40, 51

^†^Albumin at diagnosis are shown. *N* = 92, 40, 52

**Table 2:** Microbiological profiles of bronchoscopic specimens at the time of diagnosis

Bacteria	Overall (*N* = 93)	Stable group (*N* = 41)	Progressive group (*N* = 52)	*P* value
NTM species				0.404
*M. avium*	63 (67.7)	29 (70.7)	34 (65.4)	
*M. intracellulare*	29 (31.2)	11 (26.8)	18 (34.6)	
*M. avium* and *M. intracellulare*	1 (1.1)	1 (2.4)	0 (0)	
AFB smear-negative	65 (69.9)	28 (68.3)	37 (71.2)	0.765
AFB smear-positive (grade 1+)	28 (30.1)	13 (31.7)	15 (28.8)	
AFB smear-positive (grade ≥ 2+)	0 (0)	0 (0)	0 (0)	
Time to culture positive (week)	1 (1–1)	0 (0–1)	1 (0–1)	0.355
Other species				
*S. aureus*	21 (22.6)	9 (22.0)	12 (23.1)	0.897
*Veillonella species*	53 (57.0)	24 (58.5)	29 (58.5)	0.789
*Aspergillus species*	13 (14.0)	7 (17.1)	6 (11.5)	0.445
*P. aeruginosa*	8 (8.6)	3 (7.3)	5 (9.6)	0.695

NTM, nontuberculosis mycobacteria; *M. avium*, *Mycobacterium avium*

*M. intracellulare*, *Mycobacterium intracellulare*; AFB, acid-fast bacilli; *S. aureus*, *Staphylococcus aureus*; *P. aeruginosa, Pseudomonas aeruginosa.* Categorical variables are represented as absolute numbers (%). Medians (interquartile range) are used to describe non-parametric data. Categorical variables were analyzed using the Chi-squared test. Non-parametric data were analyzed by the Mann–Whitney *U* test. *P* values are for comparisons between stable group and progressive group

The radiological characteristics of the two groups are shown in Table 3. NB type was the most common radiographic pattern (98.9%), and most cases (93.5%) had middle lobe (lingula) involvement. The frequent combinations of involved lobes were upper + middle (lingula) + lower lobe in 29 (31.2%) patients, upper + middle lobe (lingula) in 24 (25.8%) patients, middle (lingula) + lower lobe in 19 (20.4%) patients, and only the middle lobe (lingula) in 15 (16.1%) patients. There was no difference in the predominant patterns on CT imaging between the stable and progressed groups (Table 3).

**Table 3:** Radiological characteristics

	Overall (*N* = 93)	Stable group (*N* = 41)	Progressive group (*N* = 52)	*P* value
FC type	1 (1.1)	0 (0)	1 (1.9)	0.372
NB type	92 (98.9)	41 (100)	51 (98.1)	0.372
Nodular/granular shadow predominant	56/92 (60.9)	28/41 (68.3)	28/51 (60.9)	0.271
Bronchiectasis predominant	24/92 (26.1)	10/41 (24.4)	14/51 (27.5)	
Consolidation predominant	12/92 (13)	3/41 (7.3)	9/51 (17.6)	
The location of the lesion				
U	3 (3.2)	1 (2.4)	2 (3.8)	0.703
M	15 (16.1)	9 (22.0)	6 (11.5)	0.175
L	0 (0)	0 (0)	0 (0)	
U + M	24 (25.8)	12 (29.3)	12 (23.1)	0.498
U + L	3 (3.2)	2 (4.9)	1 (1.9)	0.423
M + L	19 (20.4)	5 (12.2)	14 (26.9)	0.080
U + M + L	29 (31.2)	12 (29.3)	17 (32.7)	0.723
Total	93 (100)	41 (100)	52 (100)	
M + L, or U + M + L	48 (51.6)	17 (41.5)	31 (59.6)	0.082
The number of involved lobes				
1–2	32 (34.4)	14 (34.1)	18 (34.6)	0.962
3–4	47 (50.5)	21 (51.2)	26 (50.0)	0.907
5–6	14 (15.1)	6 (14.6)	8 (15.4)	0.920

FC, Fibrocavitary; NB, Nodular bronchiectatic; U, Upper lobes; M, Middle lobes; L, Lower lobes. Data are presented as *N* (%).* P* values are for comparisons between stable group and progressive group. Categorical variables were analyzed using the Chi-squared test

### Risk factors for clinical progression

**Fig. 1:**
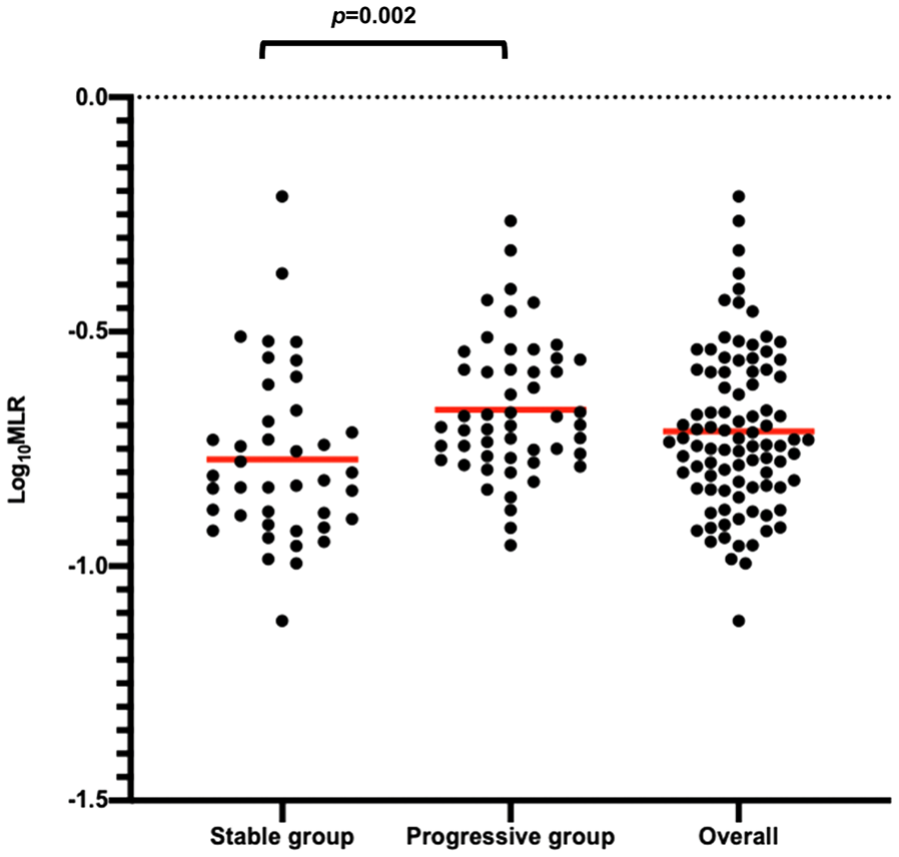
Log_10_MLR for the stable group and the progressed group. Each dot and horizontal bar represent Log_10_MLR and the mean, respectively. Student’s *t* test was performed comparing the stable and progressed groups. The Log_10_MLR is significantly lower in the stable group than in the exacerbation group (*p* = 0.002). Definitions of abbreviations: MLR, monocyte lymphocyte ratio

The progressed patients were more likely to be male (12.2% vs. 30.8%; *p* = 0.033). Monocyte counts (256 vs. 285; *p* = 0.018) and MLRs (0.15 vs. 0.20; *p* < 0.001) were higher in the progressed group than in the stable group (Table 1). The mean Log_10_MLR was lower in the stable group than in the progressed group (−0.77 vs. −0.67; *p* = 0.002) (Table 1; Fig. 1).

The remaining patients’ characteristics, including age, BMI, smoking status, comorbidities, and respiratory symptoms did not differ between the two groups. Almost half of the cases were negative for GPL-core IgA antibody (Table 1). In addition, the rate of patients who were positive for GPL-core IgA antibody did not differ between the progressed group and the stable group. No significant difference in microbiological status was found between the two groups (Table 2).

Although there were no differences between the two groups in radiological types, the location of the lesion, and the number of involved lobes (Table 3), the combination of middle lobe (lingula) and lower lobe involvement tended to be higher in the cases with clinical progression (41.5% in stable group vs. 59.6% in progressed group; *p* = 0.082) (Table 3).

On multivariate analysis, male sex, MLR ≥ 0.17, and the presence of combined lesions in the middle (lingula) and lower lobes were independent predictors of disease progression within 4 years after diagnosis (Table 4).

**Table 4:** Univariate and multivariate analyses of risk factors for progression in the study subjects

Variables	Univariate analysis		Multivariate analysis	
	OR (95% CI)	*P* value	Adjusted OR (95% CI)	*P* value
Age	1.01 (0.97–1.06)	0.572	1.00 (0.95–1.06)	0.939
Male sex	3.20 (1.06–9.67)	**0.039**	3.87 (1.09–13.67)	**0.037**
Body mass index	0.88 (0.74–1.06)	0.173		
Alb	0.47 (0.16–1.37)	0.165		
MLR ≥ 0.17	4.71 (1.92–11.52)	**<0.001**	5.03 (1.92–13.18)	**0.001**
The location of the lesion in M + L, or U + M + L	2.08 (0.91–4.79)	**0.084**	3.02 (1.15–7.94)	**0.025**

OR, Odds ratio; CI, confidence interval; MLR, Monocyte to lymphocyte ratio; U, Upper lobes; M, Middle lobes; L, Lower lobes. The age, sex, and the variables with a *P* value of less than 0.1 in the univariate analysis were entered into the multivariate logistic regression analysis. Bold indicates significance (*P* < 0.1 in the univariate analysis and *P* < 0.05 in the multivariate analysis)

### MLR for detecting progression of pulmonary MAC disease

**Fig. 2:**
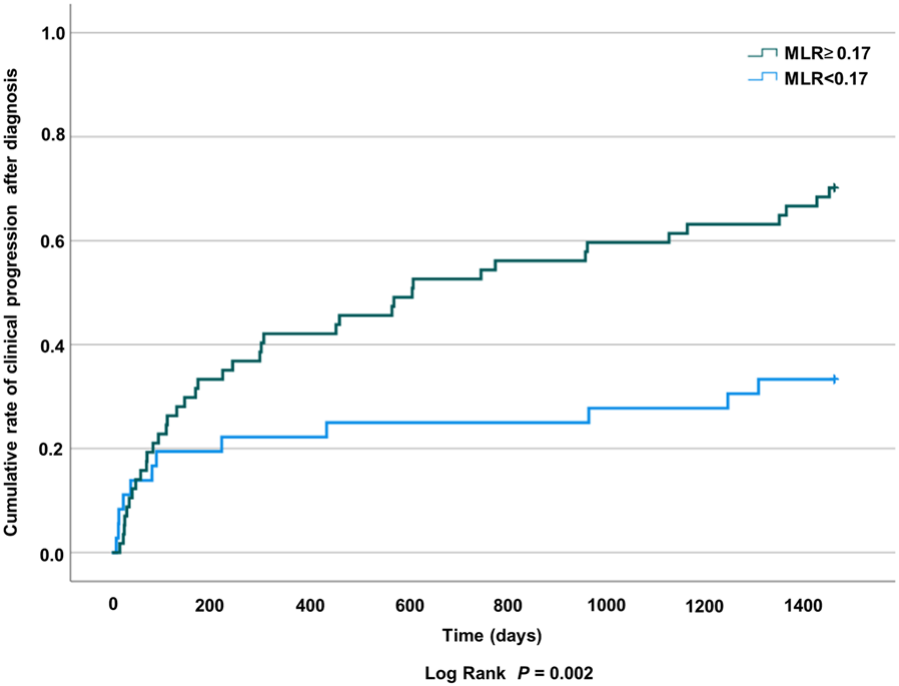
Cumulative rate of clinical progression of pulmonary MAC patients with low and high MLRs. The log-rank test shows that the cumulative rate of pulmonary MAC disease 4 years after diagnosis is significantly higher in patients with a high MLR. Definitions of abbreviations: MAC, *Mycobacterium avium* complex; MLR, monocyte lymphocyte ratio

**Fig. 3:**
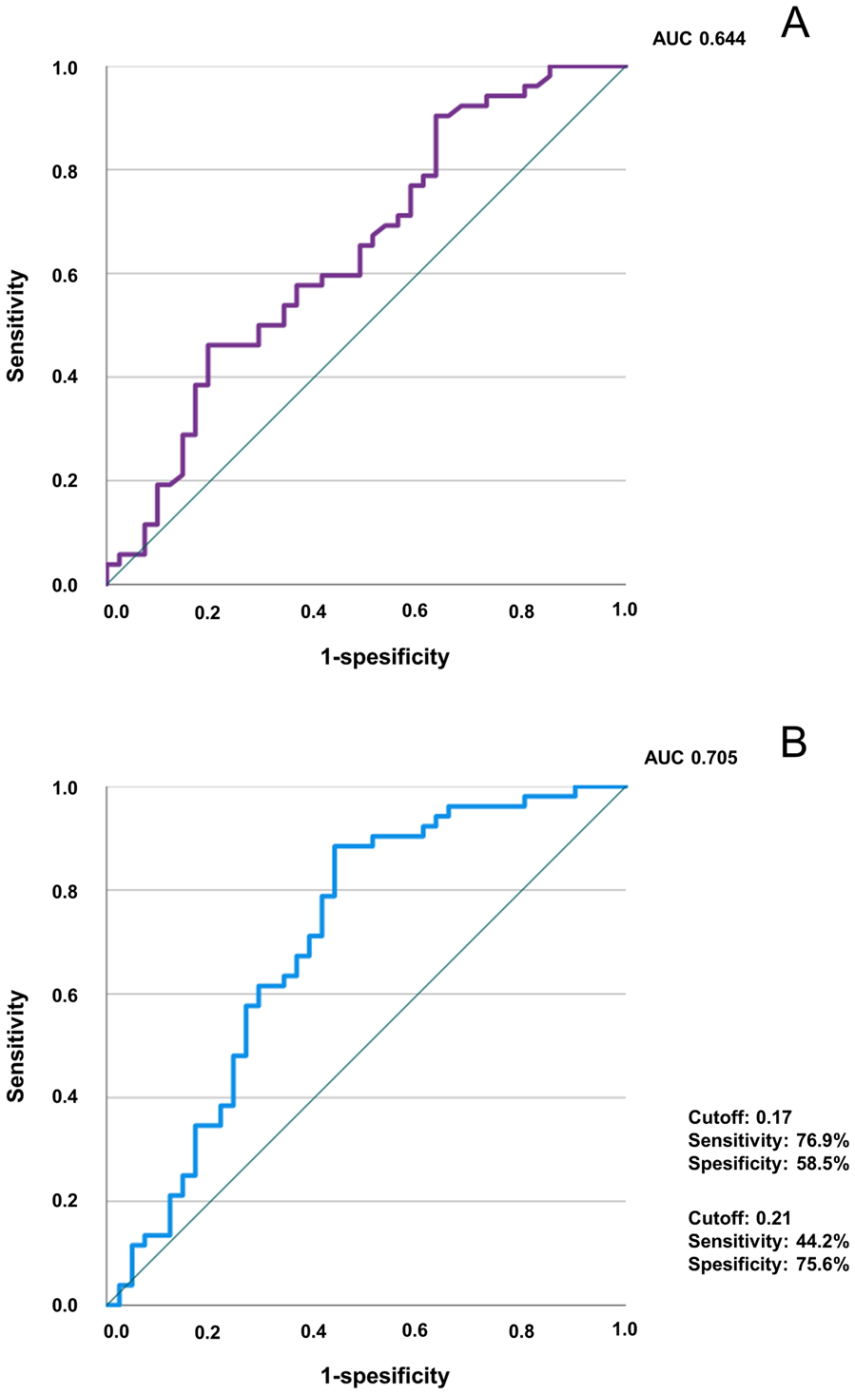
ROC curve for detecting clinical progression of pulmonary MAC disease after diagnosis. **a** ROC curve of monocyte counts, **b** ROC curve of the MLR. ROC curve of the MLR shows that the sensitivity, specificity, and area under the curve are 76.9%, 58.5%, and 0.705, respectively, at a cutoff of 0.17 for identifying progression of pulmonary MAC disease after diagnosis. With a cutoff value of 0.21, the MLR predicts progression with a sensitivity of 44.2% and specificity of 75.6%. ROC, receiver-operating characteristic; MAC, *Mycobacterium avium* complex; MLR, monocyte lymphocyte ratio

The log-rank test showed that the cumulative rate of disease progression within 4 years after diagnosis was significantly lower in patients with a lower MLR (*p* = 0.014) (Fig. 2). ROC curve analysis applied to examine the efficiency of monocyte counts and the MLR in detecting progression of pulmonary MAC disease showed areas under the ROC curve (AUC) of 0.644 and 0.705, respectively (Fig. 3A, B). The AUC for the MLR was greater than 0.7, indicating moderate accuracy for predicting progression. With a cutoff value of 0.17, the MLR predicted progression with a sensitivity of 76.9% and specificity of 58.5%. The MLR showed high specificity at a cutoff value of 0.21 (sensitivity 44.2%, specificity 75.6%, Fig. 3B).

### Comparison of CBC according to the number of involved lobes

**Fig. 4:**
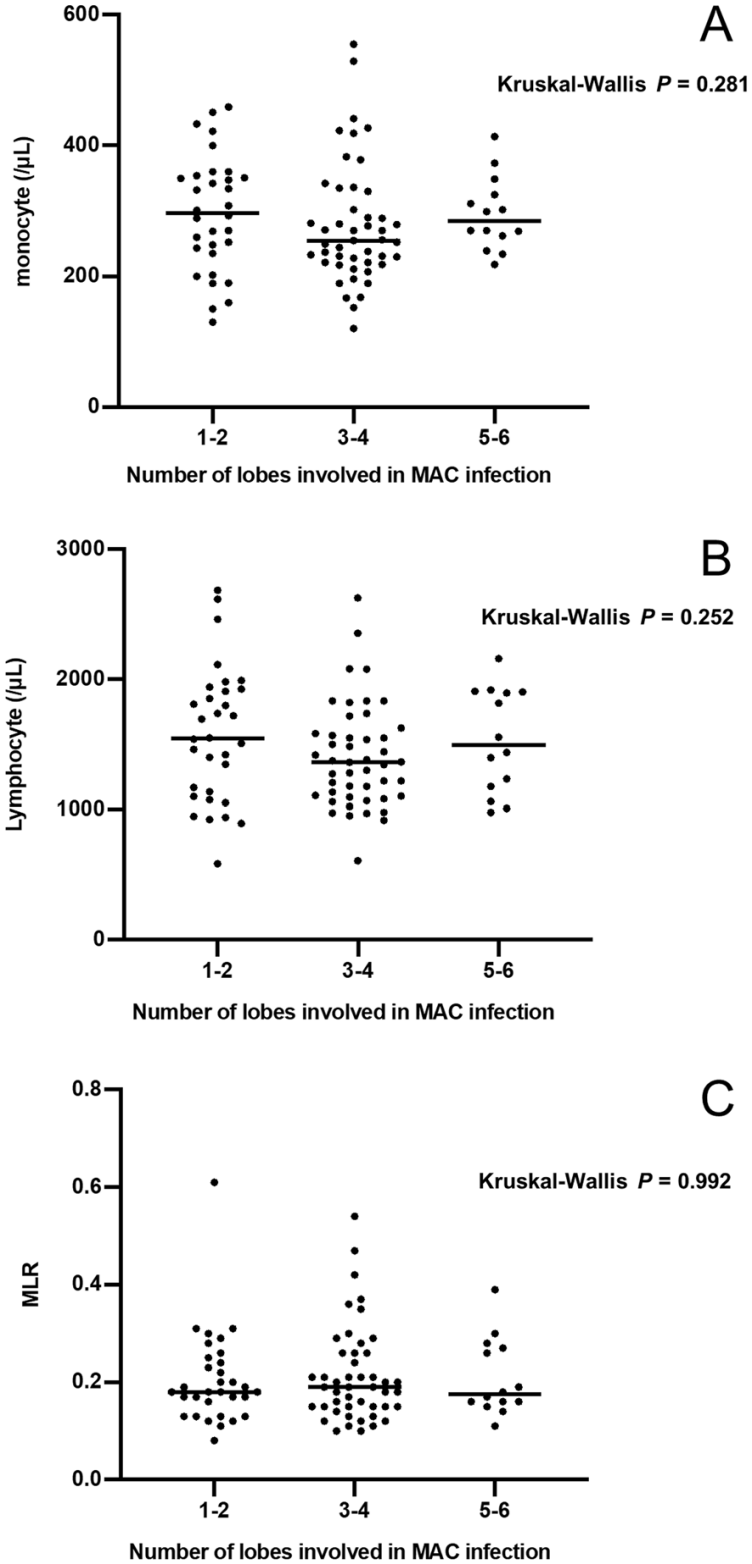
Comparison of laboratory examination results among the patients with different numbers of involved lobes. **a** Comparison of monocyte counts, **b** comparison of lymphocyte counts, **c** comparison of MLRs. Continuous variables with non-normal distributions are analyzed using the Kruskal–Wallis test. A horizontal bar indicates the median. Comparison analysis shows no differences in monocyte counts, lymphocyte counts, or MLRs among the patients with different numbers of involved lobes. MLR, monocyte lymphocyte ratio

To examine the relationship between the extent of lung lesions and leukocyte counts, the MLRs were compared among three groups according to the number of involved lobes (Fig. 4). No significant differences were found among them in lymphocytes, monocytes, and MLRs, suggesting that the numbers of monocytes and lymphocytes and the MLRs were not associated with the extent of pulmonary lesions.

## Discussion

This retrospective study investigated the risk factors for progression of pulmonary MAC disease diagnosed by bronchoscopy. More than half of the patients deteriorated clinically, and one-third had culture-positive sputum within 4 years of diagnosis. The risk factors for disease progression were found to be male sex, MLR ≥ 0.17, and the presence of combined lesions in the middle (lingula) and lower lobes. This study is the first to show a relationship between the MLR and activity of pulmonary MAC disease, although the MLR has been investigated as a biomarker in some infectious diseases, including tuberculosis [14, 15, 18–20]. The association between higher MLR values and disease progression may reflect increased monocytes or decreased lymphocytes [13, 18, 21]. A previous study showed that monocytes from individuals with high MLRs showed impaired control of mycobacterial growth and a distinctive transcriptome that may explain the associations of the MLR with tuberculosis and other inflammatory diseases [19]. Therefore, the high monocyte counts in the advanced group in this study may support the contention that monocyte dysfunction is involved in the progression of pulmonary MAC disease, and further investigation is warranted.

Positivity for GPL–IgA antibody was not associated with progression in the present study. Previous reports suggested that GPL antibody levels may be associated with disease progression [16, 22]. A retrospective study in Japan showed that reduction of GPL core antibody levels is associated with disease activity and treatment outcomes in pulmonary MAC patients [22]. In the present study, however, GPL–core IgA antibody-positivity did not differ between the progressed group and the stable group. The fact that nearly half of the cases in the present study were negative for GPL–core IgA antibodies suggests that GPL–core IgA antibody levels appear to be a poor marker for diagnosis and prediction of progression of pulmonary MAC disease without culture-positive sputum.

The association between the location of pulmonary MAC disease and disease progression has rarely been reported. Severe pulmonary MAC patients were reported to have more lesions in S2 and S6 of the lungs [23]. Furthermore, non-cystic fibrosis bronchiectasis (NCFB) is commonly observed in the middle and lower lobes of the lungs, reflecting a failure of mucociliary clearance and gravity-dependent mucus accumulation [24]. Because decreased mucociliary clearance is also important in pulmonary NTM disease [25, 26], the presence of lesions in the middle (lingula) and lower lobes may reflect much more decreased mucociliary clearance in the progressed group of the present study. A study with a larger sample size, using CT, could clarify how pulmonary MAC disease diagnosed by bronchoscopy progresses over time.

Previous studies suggested that *Pseudomonas aeruginosa* or *Aspergillus* species infections may have negative impacts on patients with pulmonary MAC disease [27–29]. Furthermore, it has been reported that bronchiectatic lesions are more common in co-infected patients than in patients with pulmonary MAC disease alone. However, neither the microbiological findings nor the bronchiectasis-predominant type in the present study affected disease status. This result may have been affected by the fact that patients with pulmonary MAC diagnosed by bronchoscopy had fewer cavities, less severe bronchiectasis, and less presence of locus minoris resistentiae [30, 31]. On the other hand, *Veillonella* species, anaerobic bacteria, were detected in almost half of the pulmonary MAC patients in both groups. This result supports the previous study showing that anaerobic bacteria were detected more frequently in patients with NTM [32].

In the current study, male sex was found to be a factor associated with a poor prognosis. In mice given intravenous *Mycobacterium intracellulare*, male mice had more extensive lesions in various organs and a higher bacterial load than female mice [33]. This study showed that the antimicrobial activity of macrophages was stronger in female mice than in male mice. In addition, despite the higher incidence of pulmonary NTM disease in females, several studies have shown a worse prognosis in males than in females [34–36], and the present findings are consistent with these previous reports.

In a previous study including patients infected with *M. kansasii* and *M. abscessus* in addition to MAC, in patients diagnosed with pulmonary NTM disease by bronchoscopy, male, BMI < 18.5 kg/m^2^, ICS user, and AFB smear ≥ grade 2 on bronchoscopic specimens were independent predictors of radiographic progression [9]. Because different types of NTM have different pathogenicity and patient backgrounds [37–39], the results of risk factors for progression may also differ according to the different types of NTM.

There are some limitations to the present study. First, this study was conducted with a small sample size at a single institution in Japan. The present findings need to be confirmed in a larger, multicenter study. Second, cases of patients who were not followed for more than 4 years after diagnosis were excluded. These might have included fatal cases or dropout cases with mild symptoms. Third, the timing of sputum examination and initiation of treatment after diagnosis depends on the attending physician’s judgment based on symptoms and radiological deterioration, and thus inter-physician bias should be considered.

## Conclusion

This single-center, retrospective, observational study identified male sex, higher MLR, and the presence of lesions in the middle (lingula) and lower lobes as the risk factors for clinical progression of pulmonary MAC patients without positive sputum cultures. Since more than half of patients with pulmonary MAC disease diagnosed by bronchoscopy progressed clinically within 4 years, careful follow-up, especially in patients with the risk factors identified in the present study, is warranted.

## Data Availability

Not applicable.

## Abbreviations

NTM: Nontuberculous mycobacteria
ATS: American Thoracic Society
ERS: European Respiratory Society
ESCMID: European Society of Clinical Microbiology and Infectious Diseases
IDSA: Infectious Diseases Society of America
AFB: Acid-fast bacilli
BMI: Body mass index
ICS: Inhaled corticosteroid
MAC: *Mycobacterium avium* Complex
CBC: Complete blood count
MLR: Monocyte to lymphocyte ratio
GPL: Glycopeptidolipid
GBT: Guideline-based therapy
CT: Computed tomography
FC: Fibrocavitary
NB: Nodular bronchiectatic
MGIT: Mycobacteria Growth Indicator Tube
BTB: Bromothymol blue
ROC: Receiver-operating characteristic
AUC: Area under the receiver-operating characteristic curve
NCFB: Non-cystic fibrosis bronchiectasis
